# Dose Finding in Oncology Trials Guided by Ordinal Toxicity Grades Using Continuous Dose Levels

**DOI:** 10.3390/e26080687

**Published:** 2024-08-14

**Authors:** Mourad Tighiouart, André Rogatko

**Affiliations:** 1 Department of Computational Biomedicine, Cedars-Sinai Medical Center, Los Angeles, CA 90069, USA; 2 Independent Researcher, 2765-399 Monte Estoril, Portugal

**Keywords:** cancer phase I trials, maximum tolerated dose, ordinal toxicity grade, proportional odds model, overdose control, continuous dose

## Abstract

We present a Bayesian adaptive design for dose finding in oncology trials with application to a first-in-human trial. The design is based on the escalation with overdose control principle and uses an intermediate grade 2 toxicity in addition to the traditional binary indicator of dose-limiting toxicity (DLT) to guide the dose escalation and de-escalation. We model the dose–toxicity relationship using the proportional odds model. This assumption satisfies an important ethical concern when a potentially toxic drug is first introduced in the clinic; if a patient experiences grade 2 toxicity at the most, then the amount of dose escalation is lower relative to that wherein if this patient experienced a maximum of grade 1 toxicity. This results in a more careful dose escalation. The performance of the design was assessed by deriving the operating characteristics under several scenarios for the true MTD and expected proportions of grade 2 toxicities. In general, the trial design is safe and achieves acceptable efficiency of the estimated MTD for a planned sample size of twenty patients. At the time of writing this manuscript, twelve patients have been enrolled to the trial.

## 1. Introduction

Phase 1 clinical trials in oncology are usually small studies to test the safety and tolerability of first-in-human cancer drugs or a combination of existing ones. These trials are generally open to a heterogeneous population of cancer patients with late-stage disease who have exhausted all standard therapy [1]. As a result, ethical issues have been raised by many authors due to targeting vulnerable patients [2,3,4], potential risks to patients with low therapeutic effects [5,6,7], and an improbable expectation of benefits from the patients’ perspectives [8,9,10,11]. However, owing to the development of targeted agents and immune checkpoint inhibitors, a recent study analyzing 465 clinical protocols of cancer phase 1 trials showed that the response rate doubled over the past two decades, reaching 18% in the last 10 years, while the risk of treatment-related deaths remained unchanged, at less than 1% [12]. In this manuscript, we are concerned with cancer phase I trials whose primary endpoint is the determination of the maximum tolerated dose (MTD) or recommended phase 2 dose (RP2D). This is defined as the dose γ that is expected to produce dose-limiting toxicity (DLT) within one or more cycles of treatment in a prespecified proportion θ of patients:

(1) P(DLT|Dose=γ)=θ.

The number of prespecified cycles of therapy to resolve the DLT status of a patient for the purpose of dose escalation and de-escalation depends on the nature of treatment and schedule of administration. For example, if only immunotherapy agents are used, then the DLT window of time to resolve toxicity is often taken as two cycles of treatment. For treatment involving radiation therapy with expected late-onset toxicity, an observation window of four to six cycles is taken instead.

The definition of DLT depends on the expected treatment-related side effects or toxicities graded according to the Common Terminology Criteria for Adverse Events (CTCAE) version 5 [13]. For safety and ethical reasons, these trials enroll successive cohorts of patients starting with the minimum dose available in the trial or a dose deemed safe by the investigator, and the dose allocated to the next cohort of patients depends on the doses and toxicity outcomes of the previously treated patients. To this end, statistical designs have been studied extensively over the last three decades and shown to be safe and efficient in estimating the MTD. These include parametric models such as the continual reassessment method (CRM) [14], escalation with overdose control (EWOC) [15,16,17], the Bayesian logistic regression model (BLRM) [18], semi-parametric design [19], and nonparametric dose finding methods including the modified toxicity probability interval (mTPI) [20], Bayesian optimal interval design (BOIN) [21], and the nonparametric overdose control method [22].

The vast majority of statistical designs for phase 1 trials that are used in practice assume that the DLT is a binary indicator of toxicity, typically taking the value 1 if a patient experiences a grade 3 or 4 non-hemathologic or grade 4 hemathologic toxicity, and zero otherwise, despite the fact that many authors extended these designs to account for multiple toxicity types and grades using ordinal toxicity and quasi-continuous grades (see, e.g., [23,24,25,26,27,28,29,30,31,32,33]). Indeed, some of these methods have shown that accounting for lower grade toxicities may result in less patients being overdosed when the true MTD is near the minimum dose level available in the trial. In this manuscript, we extend the design in [24] by allowing the MTD to be outside the dose range with positive probability a priori using the model reparameterization introduced in [34]. This design is based on the EWOC principle by guiding dose escalation using grade 2 toxicity in addition to the binary indicator of DLT. This extension was motivated by a conversation with a clinician for the purpose of designing a first-in-human trial where it is desirable to have a more careful dose escalation and there is high uncertainty regarding the maximum available dose in the trial obtained from preclinical experiments. We show that our design addresses the ethical concerns regarding dose escalation in the presence of low-grade toxicity and the absence of DLT; if a patient experiences grade 2 toxicity at the most, then the size of the dose escalation is lower relative to that which would’ve occurred had the current patient experienced a maximum of grade 1 toxicity. Moreover, the design is coherent in both escalation and de-escalation.

The article is organized as follows. In Section 2, we present the dose–toxicity model for ordinal toxicity grades and the dose escalation algorithm EWOC scheme. Properties of the design to address some of the ethical concerns in first-in-human trials are described in Section 3. In Section 4, we apply our methodology to design a real-life phase 1 trial with continuous dose levels and assess the performance of the design by simulating trials under several scenarios for the location of the true MTD and rates of grade 2 toxicity. We conclude our manuscript with a discussion in Section 5.

## 2. Materials and Methods

### 2.1. Model

Let $X_{\min}$ and $X_{\max}$ be the minimum and maximum doses available in the trial, $X_{\min}>0$. We assume that all CTCAE toxicities and their respective grades are collected by the end of the first treatment cycle so that the DLT observation window is one cycle of treatment. Following the work of [24], let G=0,1,…,4 be the maximum grade of any type of toxicity experienced by a patient by the end of cycle 1. Define

$$Y=\left\{\begin{array}{cc} 0 & \mathrm{if}G=0,1\\ 1 & \mathrm{if}G=2\\ 2 & \mathrm{if}G=3,4, \end{array}\right.$$

where the event Y=2 corresponds to a DLT. The dose–toxicity association is modeled using a known strictly increasing cumulative distribution function (cdf) F(·) as follows:

(2) $$P\left(Y\geq j|x\right)=F\left(\eta_{j}+\beta x\right),j=1,2,$$

where $x\in [X_{\min},X_{\max}]$, $\eta_{1},\eta_{2}$ are the model intercepts and β is the slope parameters of a proportional odds model. The parameters $\eta_{1},\eta_{2}$ represent the logarithm of the odds of grade 2 or more toxicity and logarithm of the odds of DLT in the absence of treatment, respectively. We further assume that the probability of DLT is a non-decreasing function of dose, i.e., β≥0. By definition, the MTD, γ, which is the parameter of interest, is the maximum dose that is expected to produce DLT in a prespecified proportion θ of patients:

(3) $$P\left(\mathrm{DLT}|x\right)=P\left(Y=2|x=\gamma \right)=F\left(\eta_{2}+\beta \gamma \right)=\theta .$$

The value of the target probability of DLT θ is set to be relatively high when the DLT is a transient, correctable, or nonfatal condition and low when it is lethal or life threatening; see [35,36,37] for examples of phase 1 trials with θ=0.1,0.25, and 0.33, respectively.

### 2.2. Likelihood and Model Reparameterization

Let $D_{n}=\left\{\left(x_{i},Yi\right),i=1,\ldots ,n\right\}$ be the data after *n* patients complete the first cycle of therapy and their toxicity status has been resolved. The likelihood for the parameters $\eta_{1},\eta_{2},\beta$ is

(4) $$\begin{array}{cc} L_{n}\left(\eta_{1},\eta_{2},\beta |D_{n}\right) & =\prod_{i=1}^{n}{\left[1-F\left(\eta_{1}+\beta x_{i}\right)\right]}^{I(Y_{i}=0)}{\left[F\left(\eta_{1}+\beta x_{i}\right)-F\left(\eta_{2}+\beta x_{i}\right)\right]}^{I(Y_{i}=1)}\\ \; & \times {\left[F\left(\eta_{2}+\beta x_{i}\right)\right]}^{I(Y_{i}=2)}, \end{array}$$

where I(·) is the indicator function.

We reparameterize model (2) in terms of parameters clinicians can easily interpret. Unlike the reparameterization in [24], which assumes that the MTD is in $\left[X_{\min},X_{\max}\right]$ with prior probability 1, we adopt the reparameterization in [34], which allows for a positive prior probability that γ is outside the dose range $\left[X_{\min},X_{\max}\right]$. Specifically, let $\rho_{0}$ be the probability of DLT at $X_{\min}$, $\rho_{1}$ be the probability of grade 2 or more toxicity at $X_{\min}$, and $\rho_{2}$ be the probability of DLT at $X_{\max}$. It follows that

(5) $$\left\{\begin{array}{cc} \eta_{1}= & F^{-1}\left(\rho_{1}\right)-\left(F^{-1}\left(\rho_{2}\right)-F^{-1}\left(\rho_{0}\right)\right)\left(X_{\min}/\left(X_{\max}-X_{\min}\right)\right)\\ \eta_{2}= & F^{-1}\left(\rho_{2}\right)-\left(F^{-1}\left(\rho_{2}\right)-F^{-1}\left(\rho_{0}\right)\right)\left(X_{\max}/\left(X_{\max}-X_{\min}\right)\right)\\ \beta = & \left(F^{-1}\left(\rho_{2}\right)-F^{-1}\left(\rho_{0}\right)\right)/\left(X_{\max}-X_{\min}\right). \end{array}\right.$$

We standardize the doses to be in the interval [0,1] using the transformation $h\left(u\right)=\left(u-X_{\min}\right)/\left(X_{\max}-X_{\min}\right)$ and define

(6) $$\begin{array}{cc} F_{1}\left(\rho_{0},\rho_{1},\rho_{2};x\right)= & F\left[F^{-1}\left(\rho_{1}\right)+\left(F^{-1}\left(\rho_{2}\right)-F^{-1}\left(\rho_{0}\right)\right)x\right]\\ F_{2}\left(\rho_{0},\rho_{1},\rho_{2};x\right)= & F\left[F^{-1}\left(\rho_{0}\right)+\left(F^{-1}\left(\rho_{2}\right)-F^{-1}\left(\rho_{0}\right)\right)x\right]. \end{array}$$

It follows that the likelihood of the reparameterized model is

(7) $$\begin{array}{cc} L_{n}\left(\rho_{0},\rho_{1},\rho_{2}|D_{n}\right) & =\prod_{i=1}^{n}{\left[1-F_{1}\left(\rho_{0},\rho_{1},\rho_{2};x_{i}\right)\right]}^{I(Y_{i}=0)}{\left[F_{1}\left(\rho_{0},\rho_{1},\rho_{2};x_{i}\right)-F_{2}\left(\rho_{0},\rho_{1},\rho_{2};x_{i}\right)\right]}^{I(Y_{i}=1)}\\ \; & \times {\left[F_{2}\left(\rho_{0},\rho_{1},\rho_{2};x_{i}\right)\right]}^{I(Y_{i}=2)}. \end{array}$$

### 2.3. Prior Specification

The monotonicity assumption of the dose–toxicity relationship implies that $0\leq \rho_{0}\leq \rho_{2}\leq 1$. Furthermore, the proportional odds model assumption (2) implies that $\eta_{2}\leq \eta_{1}$. Using (5), this leads to $0\leq \rho_{0}\leq \rho_{1}\leq 1$. We therefore specify a prior distribution $g(\rho_{0},\rho_{1},\rho_{2})$ as follows: $\rho_{1},\rho_{2}$ are independent, $\rho_{j}∼\mathrm{beta}\left(a_{j},b_{j}\right),j=1,2$ and given $\left\{\rho_{1},\rho_{2}\right\}$, $\rho_{0}/\min\left(\rho_{1},\rho_{2}\right)∼\mathrm{beta}\left(a_{0},b_{0}\right)$. Using Bayes’ rule, the posterior distribution of the model parameters is

(8) $$\pi \left(\rho_{0},\rho_{1},\rho_{2}|D_{n}\right)\propto L\left(\rho_{0},\rho_{1},\rho_{2}|D_{n}\right)\times g\left(\rho_{0},\rho_{1},\rho_{2}\right).$$

Features of this posterior distribution can be estimated using JAGS [38].

### 2.4. Trial Design

In this section, we describe the general EWOC design for continuous dose levels. The criteria for dose allocation to successive cohorts of patients is based on controlling the posterior probability that the MTD γ exceeds the recommended dose level for the next cohort of patients.

The parameter of interest γ in the standardized scale can be obtained from (3):

(9) $$\gamma =\frac{F^{-1}\left(\theta \right)-F^{-1}\left(\rho_{0}\right)}{F^{-1}\left(\rho_{2}\right)-F^{-1}\left(\rho_{0}\right)}\;\;I\left(\gamma >\frac{-X_{\min}}{X_{\max}-X_{\min}}\right),$$

where I(·) is the indicator function. The lower bound $l_{b}=-X_{\min}/\left(X_{\max}-X_{\min}\right)$ results from the transformation h(·) and the fact that unstandardized doses *x* are positive.

Let *n* be the maximum number of patients to be enrolled in the trial. The first cohort of patients receive the dose $x=X_{\min}$. Denote by $\Pi_{k}\left(\gamma \right)=\Pi \left(\gamma |D_{k}\right)$ the marginal posterior cdf of the MTD, k=1,…,n−1. The next cohort of patients receive the dose $x_{k+1}=\Pi_{k}^{-1}\left(\alpha \right)$. In other words, the dose allocated to the next patient(s) is chosen such that the posterior probability that it exceeds the MTD, given the current data, is bounded by α. This is known as the overdose protection property of EWOC, where at each dose allocation stage of the trial, we seek a dose that controls the posterior probability of exposing patients to toxic dose levels. At the end of the trial, we estimate the MTD as the median of the posterior distribution of γ, $\pi (\gamma |D_{n}).$ Other design considerations such as size of the cohorts, size of the dose escalation, and stopping rules for safety are described in the phase 1 trial example of DZ-002.

## 3. Design Properties

The design described in Section 2.4 has the following properties:

(i) Overdose Control: Each time patients are ready to be enrolled to the trial, we seek a dose to allocate to these patients while controlling the posterior probability of exposing them to toxic dose levels.
(ii) Toxicity-Dependent Dose Escalation: If the maximum grade of toxicity experienced by patient *k* is grade 2, then the dose allocated to patient k+1 is lower than the dose that would have been given to patient k+1 had the maximum grade of toxicity experienced by patient *k* been grade 0 or 1.
(iii) Coherence: If patient *k* treated at dose level $x_{k}$ experiences DLT, then the dose $x_{k+1}$ allocated to patient k+1 satisfies $x_{k+1}\leq x_{k}$. Similarly, if patient *k* does not experience DLT and has no grade 2 toxicity, then the dose $x_{k+1}$ allocated to patient k+1 satisfies $x_{k+1}\geq x_{k}$.

Property (i) is the overdose protection defining characteristic of EWOC and was first introduced in [15,39]. Under the one-parameter logistic link function $F\left(u\right)={\left(1+e^{-u}\right)}^{-1}$ and the assumption of bounded support for the MTD γ a priori, ref. [39] showed that the sequence of doses generated by the EWOC algorithm minimizes the amount by which patients are underdosed and converges to γ in probability. Property (ii) implies a cautious dose escalation and is particularly appealing for drugs used in first-in-human trials; if a patient experiences grade 2 toxicity, then it is ethical not to increase the dose by the same amount had this patient exhibited grade 1 toxicity at the most. The last property is the coherence principle introduced in [40], which states that it is ethical not to increase a dose for the next patient if the previously treated patient exhibited DLT when given the same dose level. Properties (ii) and (iii) are summarized in the following theorems.

**Theorem 1.**  *Let $D_{k}=\left\{\left(x_{i},Y_{i}\right),i=1,\ldots ,k\right\}$ be the data on the first k patients according to the EWOC algorithm and $D_{k}^{\prime }=D_{k-1}\cup \left\{\left(x_{k},Y_{k}^{\prime }\right)\right\}$. Let $x_{k+1}=\Pi_{k}^{-1}\left(\alpha |D_{k}\right)$ and $x_{k+1}^{\prime }=\Pi_{k}^{-1}\left(\alpha |D_{k}^{\prime }\right)$, where $\Pi_{k}\left(\cdot |D_{k}\right)$ is the marginal posterior cdf of the MTD γ. Suppose that for all $x\in [X_{\min},X_{\max}]$ and all $\left(\rho_{0},\rho_{1}\right),0\leq \rho_{0}\leq \rho_{1}\leq 1,\left(F_{1}-F_{2}\right)/\left(1-F_{1}\right)$ is monotonically increasing in $\rho_{2}$. Then, $x_{k+1}^{\prime }\geq x_{k+1}$ whenever $Y_{k}^{\prime }=0$ and $Y_{k}=1.$*

**Theorem 2.**  *Suppose that for all $x\in [X_{\min},X_{\max}]$ and all $\left(\rho_{0},\rho_{1}\right),0\leq \rho_{0}\leq \rho_{1}\leq 1,F_{1}$ and $F_{2}$ are monotonically increasing in $\rho_{2}$. Let $x_{k}$ be the dose allocated to patient k and $Y_{k}$ be the corresponding toxicity response. Then, if $Y_{k}=2$, the dose $x_{k+1}$ allocated to the next patient satisfies $x_{k+1}\leq x_{k}$. Furthermore, if $Y_{k}=0$, then $x_{k+1}\geq x_{k}$.*

The proofs of theorems 1 and 2 are similar to the proofs in [24] since (9) implies that γ is a decreasing function of $\rho_{2}$ for fixed $\rho_{0}$ and the assumption of a bounded support for the MTD γ in [24] was not used in the proof of those theorems.

## 4. Application to Real-Life Example

### 4.1. DZ-002 Phase 1 Trial

The methodology was applied to design and conduct a phase 1 trial using the drug DZ-002 in patients with advanced solid malignancies and lymphoma (see ClinicalTrials.gov NCT04970992). The active ingredient of DZ-002 is DZ-SIM, a heptamethine cyanine dye (HMCD)–simvastin conjugate aimed at specifically targeting cancer cells that have demonstrated superior cytotoxicity compared to SIM both in tumor cell cultures and xenograft models. The starting dose of DZ-002 is $X_{\min}=0.8$ mg/kg IV, based on animal toxicity studies. The doses for subsequent patients will be based on the EWOC algorithm, taking into account grade 0 to 4 toxicities encountered in previously enrolled patients. The maximum planned dose will not exceed $X_{\max}=15$ mg/kg IV. The MTD of DZ-002 is defined as the dose such that the probability of DLT at the MTD is θ=0.33. We used the logistic function $F\left(u\right)={\left(1+e^{-u}\right)}^{-1}$ as the link function. It is easy to verify that this link function satisfies the monotonicity condition of theorem 1. The hyperparameters defining the prior distributions are $a_{j}=b_{j}=1,j=0,1,2.$ The first cohort of up to 3 patients receives the minimum dose of 0.8 mg/kg and the dose for the subsequent cohort is determined according to the EWOC algorithm. Specifically, the dose for each subsequent cohort is determined so that, based on all available data, the posterior probability that the dose exceeds the MTD γ is equal to a prespecified feasibility bound, α. The feasibility bound starts at α=0.1, and increases to a maximum value of 0.5 by increments of 0.05 whenever a new dose allocation is computed, where this value is a compromise between the therapeutic aspect of the agent and its toxic side effects. Chu et al. [41] showed that in general, this design provides better protection in limiting higher doses for patients compared with four versions of CRM designs with a similar convergence rate. Note that the toxicity-dependent dose escalation condition (ii) in Section 3 will be satisfied when the feasibility bound α reaches 0.5 and may hold for α<0.5. If the EWOC algorithm recommends a dose lower than $X_{\min}=0.8$ mg/kg, then 0.8 mg/kg is assigned to the next cohort of patients. Similarly, if the algorithm recommends a dose above $X_{\max}=15$ mg/kg, then 0.8 mg/kg is assigned to the next cohort of patients. For ethical considerations, at any time during the trial, a patient may be enrolled if there are no more than two patients with unresolved DLT status in the study. In other words, if there are three patients under treatment who did not experience DLT and have not finished their cycle 1 therapy, then another patient cannot be enrolled. Further, the clinical investigator imposes a restriction on the increments of any dose escalation. Specifically, the increment of any dose escalation is restricted not to exceed 20% of the dose range (0.8, 15). The Food and Drug Administration (FDA) further imposed that dose escalation increments cannot exceed 2-fold of the current dose. In essence, our escalation scheme is more conservative than the FDA’s recommendation for doses above 2.8 mg/kg IV. The trial plans to enroll a maximum of n=20 patients. This study will be terminated if the posterior probability of the DLT at the minimum dose that exceeds θ is 0.8 or more. Furthermore, this study will be terminated if the posterior probability of the DLT at the maximum available dose that is less than θ is 0.9 or more. In this case, the recommended MTD is the maximum dose of 15 mg/kg. At the end of the study, the MTD will be estimated as the minimum of the median of the posterior distribution of the MTD, given the data and 15 mg/kg.

**Figure 1:**
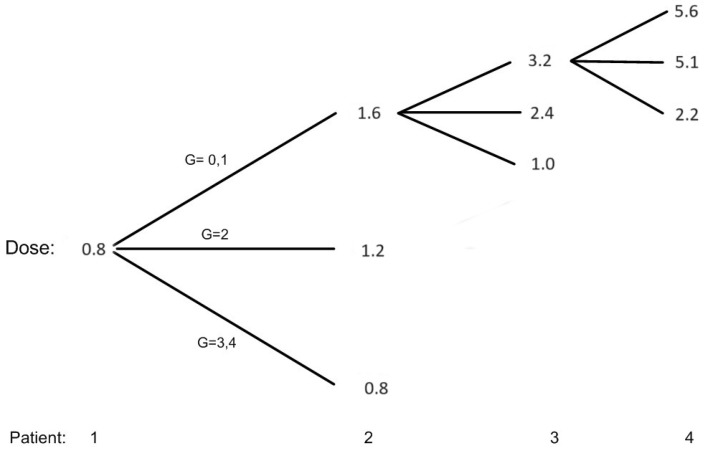
Possible dose allocation for patients 1 and 2 and selected situations for patients 3 and 4. G = 3, 4 corresponds to DLT.

Figure 1 shows all the possible dose assignments to the first two patients and selected situations for patients 3 and 4, assuming that we treat successive cohorts of one patient and the toxicity status of a patient has been resolved before enrolling the next patient. The first patient is given a minimum dose of 0.8 mg/kg, and if this patient does not have grade 2 or more toxicity, patient # 2 will be given a dose of 1.6 mg/kg. The EWOC algorithm recommends a dose much higher than 1.6 mg/kg, but this dose satisfies the FDA 2-fold restriction. If the maximum grade of toxicity for patient # 1 is 2 (G=2), than patient # 2 is assigned 1.2 mg/kg. If patients # 1, 2, and 3 do not experience grade 2 or more toxicities, then patient # 4 will be assigned 5.6 mg/kg according to the EWOC algorithm. On the other hand, if patient # 2 is treated with 1.6 mg/kg and experiences DLT, then the dose for patient # 3 decreases to 1.0 mg/kg according to the EWOC algorithm. This figure also shows that the next dose increment for a patient who experienced a maximum of grade 2 toxicity is smaller than the dose increment had this patient experienced no grade 2 or more toxicity. Furthermore, for doses greater than the minimum dose, the dose for the next patient is de-escalated form the current dose if the current patient experiences a DLT.

### 4.2. Design Operating Characteristics

We evaluate the performance of this design under three scenarios for true MTD γ and two values of $\rho_{1}$, the probability of grade 2 or more toxicity at $X_{\min}$ (see Table 1). An additional scenario # 7 was requested by the FDA. These scenarios reflect different locations for the true MTD in the interval (0.8,15.0) mg/kg and rates of grade 2 or more toxicities. The sample size used is n=20 and M=2000 trial replicates were simulated. DLTs and grade 2 or more toxicities were generated from the proportional odds model with logistic link function $F\left(u\right)={\left(1+e^{-u}\right)}^{-1}$ so that the true and working models were the same. Operating characteristics under model misspecification were studied in [24] in the case of bounded support for the MTD γ and Appendix A. Successive cohorts of one patient were treated starting with the minimum dose of 0.8 mg/kg and restrictions on dose escalation increments by the investigators and the FDA were implemented.

Table 1 gives the operating characteristics in terms of safety and efficiency of the estimated MTD. The estimated MTD is obtained by averaging the 2000 estimated MTDs, and the average bias is across all simulated trials. The percent selection column is defined as the percent of trials that recommend a dose with probability of DLT within 10% of the target probability of DLT θ=0.33. The average DLT rate is around the target probability of DLT θ=0.33 in all scenarios except when the true MTD is near the maximum dose (scenarios 3 and 6). In these cases, the average DLT rate is about 10% lower than the target. The percent of trials with an excessive rate of DLT, defined as more than an absolute 10% above the target, is small in all scenarios except when the true MTD is near the minimum dose; when the true MTD is 4.53 mg/kg, the estimated probability that a prospective trial results in a DLT rate above θ+0.1 is about 0.23. In general, the trial is safe when the true MTD is not located near the minimum dose. This is consistent with model-based and nonparameteric designs of cancer phase 1 trials. With respect to efficiency, in all scenarios, the estimated MTD is reasonably close to the true MTD and the percent selection is very high when the true MTD is near the maximum dose (scenarios 3 and 6), i.e., scenarios that are expected by the clinician. We also note the contribution of using grade 2 toxicity to guide the dose escalation in addition to DLT. For instance, scenarios 4 and 7 are cases where the true MTD is low. However, the percent selection for scenario 7 is 74% compared to 65% for scenario 4. This is due to the small probability of grade 2 or more DLT for scenario 7 $\left(\rho_{1}=0.3\right)$ compared to $\rho_{1}=0.8$ for scenario 4. With a high rate of grade 2 or more toxicity, dose escalation is slower.

**Figure 2:**
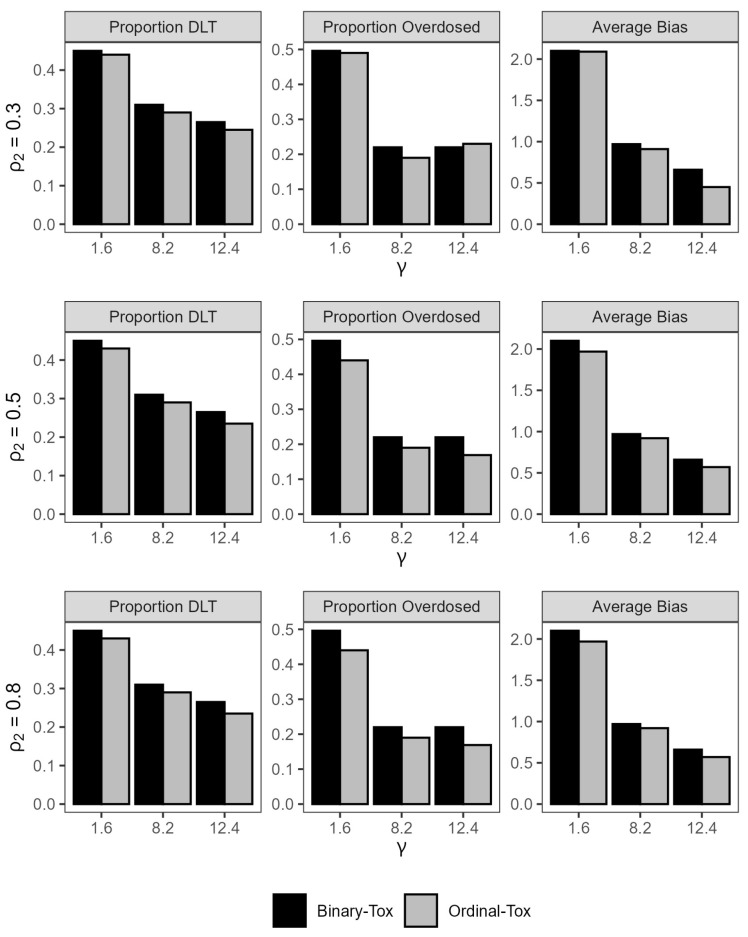
Summary statistics for trial safety and efficiency under ordinal toxicity and binary toxicity models under all scenarios.

The gray bar-plots in Figure 2 show the impact of the probability of grade 2 or more toxicity $\rho_{2}$ on the safety of the trial and efficiency of the estimated MTD. For fixed values of the true MTD, the maximum change in the absolute average bias is 0.15 mg/kg, which is negligible for practical purposes, as $\rho_{2}$ varies from 0.3 to 0.8. When the true MTD is small (1.6 mg/kg), the average DLT rate decreases by an absolute 2% when $\rho_{2}$ increases from 0.3 to 0.8. For a large value of the MTD (12.4 mg/kg), the percent of patients that were overdosed across all trials decreases by an absolute 6% from 23% to 17% when $\rho_{2}$ increases from 0.3 to 0.8. Here, a dose *x* is defined as an overdose if $x>x^{*}$, where $x^{*}$ is defined as the dose for which $P(Y=1|x^{*})=\theta +0.1$. This observation illustrates the importance of including grade 2 toxicities in the model for treatments that are expected to produce a high proportion of these toxicities. The differences may be larger for larger sample sizes. In conclusion, the design is reasonably safe and efficient for a sample size of n=20.

### 4.3. Comparing Ordinal Toxicity to Binary Toxicity

We further compared the performance of the proportional odds model (2) (ordinal toxicity) to the traditional model that does not account for grade 2 toxicity (binary toxicity). Specifically, the binary toxicity model assumes that

$$Y=\left\{\begin{array}{cc} 0 & \mathrm{if}G=0,1,2\\ 1 & \mathrm{if}G=3,4, \end{array}\right.$$

where the event Y=1 corresponds to a DLT. The dose–toxicity association is modeled using a known strictly increasing cumulative distribution function (cdf) F(·), as follows:

(10) P(Y=1|x)=F(η+βx).

The performance of these models was assessed by simulating 2000 trail replicates using the same settings of Section 4.2 under three scenarios for the true MTD γ=1.6,8.2,12.4 mg/kg and three probabilities of grade 2 or more toxicity $\rho_{2}=0.3,0.5,0.8$ for a total of nine scenarios. In addition to the average DLT rate and absolute average bias across all trials, we also compared the models with respect to the proportion of patients that were overdosed.

Figure 2 shows that the average rate of DLTs is always smaller for the model that accounts for a grade 2 toxicity relative to the binary toxicity model across all scenarios with the largest difference of 3% achieved when the true MTD is near the minimum dose and the true probability of grade 2 or more toxicity is high $(\rho_{2}=0.8).$ Similarly, the proportion of patients that were overdosed is lower when we account for a grade 2 toxicity in eight out of the nine scenarios, with the highest difference achieved when the true MTD is 1.6 mg/kg and $\rho_{2}$ is high. As for efficiency of the estimated MTD, the absolute average bias is uniformly smaller for the ordinal toxicity model relative to the binary toxicity model across all scenarios. These results are generally consistent with the findings in [24].

## 5. Discussion

In this manuscript, we described a Bayesian adaptive scheme to design and conduct a first-in-human dose finding trial in oncology. To the best of our knowledge, this is the first application of a real phase 1 trial that uses continuous dose levels and is guided by DLT as well as grade 2 toxicity outcomes. Unlike phase 1 trials that use accelerated titration [42] for the first few patients in the trial, our design uses lower-grade toxicity outcomes throughout the trial. The grouping of toxicities into three categories was selected based on the discussion with the clinician, who felt that a grade 2 toxicity should inform us that the dose causing this type of toxicity is an indication that we may be close to reaching the MTD. Comparison of the performance of this model with the one that retains the original toxicity grades as a function of sample size will be investigated in future work. The trial is ongoing and at the time of writing this manuscript, about 12 patients have been enrolled to the trial. Our approach is appropriate for handling the ethical concern regarding dose escalation in the presence of low-grade toxicity and the absence of DLT. For instance, if a patient experiences grade 2 toxicity at the most, then it is ethical not to escalate the dose for the next patient by the same amount as the one had the current patient experienced a maximum of grade 1 toxicity. This is particularly important for first-in-human trials where a more careful dose escalation scheme is warranted. In addition, the coherence in escalation and de-escalation principles introduced by the authors of [40] holds under the proportional odds model assumption (2). Finally, an additional restriction on the size of dose escalation is imposed whenever the EWOC algorithm recommends an increase in dose for the next cohort of patients. The clinician selected an increase of no more than 20% of the dose range, which is more conservative than the 2-fold recommendation from the FDA for doses above 2.8 mg/kg. Our design achieves reasonable operating characteristics under the proportional odds model, a relatively small sample size, and three unknown parameters $\rho_{0},\rho_{1},\rho_{2}$ with vague prior distributions. In practice, it is important to calibrate the prior hyperparameters following conversations with the clinician regarding the uncertainty of the location of the true MTD. For instance, expert opinion from the clinician regarding the a priori probabilities that the true MTD is above the maximum and below the minimum doses, and effective sample size may be specified to determine the hyperparameters.

## Figures and Tables

**Table 1:** Operating characteristics under seven scenarios.

Scenarios		Safety		Efficiency		
$\#:(\rho_{0},\rho_{1},\rho_{2})$	**True MTD (mg/kg)**	**Ave DLT Rate**	**% Trials with** **DLT Rate >** θ+0.1	**Estimated MTD**	**Average Bias**	**Percent Selection**
1: (0.12, 0.50, 0.95)	4.53	0.38	22%	5.04	1.30	65%
2: (0.03, 0.50, 0.90)	7.76	0.33	5%	7.94	0.98	64%
3: (0.01, 0.50, 0.40)	14.02	0.22	0.0%	13.6	−0.36	78%
4: (0.12, 0.80, 0.95)	4.53	0.38	23%	5.09	1.35	65%
5: (0.03, 0.80, 0.90)	7.76	0.34	5%	7.96	0.99	63%
6: (0.01, 0.80, 0.40)	14.02	0.23	0%	13.72	−0.33	80%
7: (0.20, 0.30, 0.70)	5.21	0.35	12%	6.56	1.98	74%

**Figure A1:**
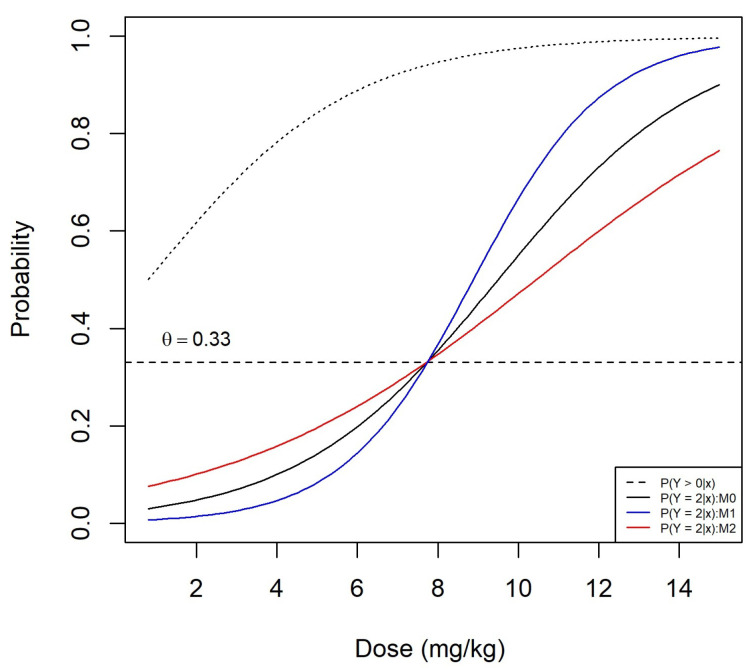
Dose–toxicity relationship under the proportional odds model (black solid curve) and non-proportional odds models $M_{1}$ and $M_{2}$ when the true MTD is γ=7.76 mg/kg. The black dashed curve corresponds to the probability of grade 2 or more toxicity.

## Data Availability

The original contributions presented in the study are included in the article, further inquiries can be directed to the corresponding author.

## Appendix A. Model Robustness

We assessed model misspecification with respect to the proportional odds model assumption (2) by generating the toxicity responses from a non-proportional odds model:

(A1) $$P\left(Y\geq j|x\right)=F\left(\eta_{j}+\beta_{j}x\right),j=1,2.$$

Figure A1 shows the probability of DLT as a function of dose for the true proportional odds model $M_{0}$ shown by the black solid curve and the two misspecified non-proportional odds models $M_{1}$ and $M_{2}$ shown by the blue and red curves, respectively. The corresponding model parameters $\alpha_{j},\beta_{j},j=1,2$ are shown in Table A1. These parameters have been selected so that the three models have the same MTD γ=7.76 mg/kg. The probability of grade 2 or more toxicity as a function of dose is shown by a dashed black curve. The operating characteristics were derived under the same settings of Section 4.2 using the logistic link function $F\left(u\right)={\left(1+e^{-u}\right)}^{-1}$ in Equation (A1).

Table A1 shows that the average DLT rate across all 2000 simulated trials is 0.33 for models $M_{0}$ and $M_{1}$ and 0.32 for model $M_{2}$. The maximum difference in the percent of trials with an excessive rate of DLT between the true model $M_{0}$ and misspecified models is 2%. Similarly, the largest absolute difference in the average bias between the true and misspecified models is 0.25 mg/kg. Lastly, the percent selection of the misspecified models are within 3% of the true model $M_{0}$. We conclude that the proportional odds model are fairly robust to small deviations from the true model around the MTD.

**Table A1:** Operating characteristics under seven scenarios.

Scenarios		Safety		Efficiency		
**Model**	**True MTD (mg/kg)**	**Ave DLT Rate**	**% Trials with** **DLT Rate >** θ+0.1	**Estimated MTD**	**Average Bias**	**Percent Selection**
$M_{0}$ $\left(\eta_{1},\eta_{2},\beta \right)$ (0,−3.47,5.67)	7.76	0.33	5%	7.94	0.98	64%
$M_{1}$ $\left(\eta_{1},\eta_{2},\beta_{1},\beta_{2}\right)$ (0,−3.47,5.67,8.78)	7.76	0.33	2.0%	7.79	0.82	61%
$M_{2}$ $\left(\eta_{1},\eta_{2},\beta_{1},\beta_{2}\right)$ (0,−3.47,5.67,3.68)	7.76	0.32	3.3%	8.19	1.23	67%
